# Evaluation of Health Education Events With a Peer-to-Peer Component in Public High Schools

**DOI:** 10.1177/15248399241258462

**Published:** 2024-06-19

**Authors:** Tai Metzger, Alex Zepeda, Naomi Wilcox

**Affiliations:** 1 Oakland University William Beaumont School of Medicine, Rochester, MI, USA; 2 University of California, Los Angeles, Los Angeles, CA, USA; 3 The Los Angeles Trust for Children’s Health, Los Angeles, CA, USA

**Keywords:** health education, information dissemination, school health, child/adolescent health, mental health, health promoting schools framework, peer education, surveys

## Abstract

Despite many improvements in childhood health outcomes, many children experience chronic health conditions and engage in unhealthy behaviors that can negatively affect the rest of their lives. School-based health education is one strategy to promote healthier lifestyles among children. Although health education is very prevalent in the United States, the methods used for this education are not well studied. Health education events such as voluntary tabling events (booths) were held by peer student educators and adult allies from the LA Trust for Children’s Health. This program evaluation studied the effects of voluntary peer-to-peer health education tabling events and a more traditional mandatory school-based health presentation. We analyzed survey responses from 343 high school students who attended 19 health education events at 8 public high schools in Los Angeles County. The results showed that health education tabling events at schools were viewed positively by students, allowing the vast majority to report learning useful information. The impact of the tabling events varied somewhat between grade levels and health topics. These findings can assist schools, health organizations, and health care providers in tailoring their programming to better disseminate health education materials and information, as well as understanding which health topics are interesting to students.

## Background

In recent years, there has been significant attention given to the role of schools in promoting public health. Prior research has shown the importance of studying the intersections between health and education. The health behaviors that are developed during childhood often carry on into adulthood, meaning that the habits cultivated as a child have lifelong consequences (Weber-Rajek et al., 2017). In addition, while great advances have been made in some areas of childhood public health, chronic conditions, such as obesity, diabetes, and asthma, have increased in children (Perrin et al., 2007). Furthermore, children who are from disadvantaged backgrounds are more likely to have poorer health and worse educational outcomes (Bradley & Corwyn, 2002). Thus, there exists a cycle in which poor health results in worsened educational outcomes, which then affects socioeconomic status (SES), which negatively impacts health (Allison et al., 2019; Raghupathi & Raghupathi, 2020). One strategy to combat this cycle is to improve childhood health through educational resources (Gupta et al., 2005).

This evaluation relies on the “Health Promoting Schools Framework” (HPS) developed by the World Health Organization (WHO) in 2014 (Langford et al., 2014). However, the HPS does not emphasize student-led or peer-to-peer health education, a key aspect of our study. Prior research has demonstrated that schools are effective settings for health education (Pulimeno et al., 2020) because they are already focused on providing education, so health education fits well into the already existing frameworks for the dissemination of knowledge. The literature also shows that adolescents have gaps in knowledge about health topics and need more education. For instance, one study on mental health knowledge among high schoolers found that students did not recognize depression and anxiety, so interventions are necessary to increase mental health literacy and increase the likelihood of seeking professional help for mental health issues (Coles et al., 2016). This finding is especially alarming, given the high rates of poor mental health among this age group (Oliveira et al., 2022). In regard to sexual health, interventions for adolescents have shown success in increasing knowledge about reproductive health (Salam et al., 2016). In particular, this study found that reproductive health education was able to improve sexual knowledge and decrease teenage pregnancy.

Peer-to-peer education programs pose an exciting method of improving health education among students. A global systematic review of the effectiveness of school-based peer education interventions concluded that peer education may be a promising strategy for improving health in schools, especially for increasing health-related knowledge (Dodd et al., 2022). However, this review found mixed effectiveness for some interventions, especially in regard to health behavior changes, and the authors highlighted the need for more evaluations of peer-led interventions. Peer-to-peer education in the health topics of substance use, mental health, and disease prevention have shown modest improvements in health knowledge and/or behavior among students (Acemoglu et al., 2011; Mall & Bhagyalaxmi, 2017; Parikh et al., 2018). Other studies have evaluated the effectiveness of a peer-to-peer sexual health education program and found that the students were able to improve their peers’ knowledge and skills on this topic (Layzer et al., 2017; Rotz et al., 2018). In terms of lifestyle improvements more generally, a school-based intervention for nutrition and exercise that had a peer-led education component improved long-term BMI among obese students (Bogart et al., 2016). Notably, one study of a mental health intervention program found that there was a positive behavior change among the peer educators rather than the peer learners (Wyman et al., 2010). This suggests that an added benefit of peer-to-peer education could be improvements among those involved in delivering the education itself.

Various types of health information dissemination have been previously studied. The literature suggests that frequency, intensity, and follow-up time are important factors for the effectiveness of health information dissemination (Chapman et al., 2020). Information leaflets and technology/internet-based dissemination methods have been found to be inexpensive ways to transmit information and improve knowledge and satisfaction (Sustersic et al., 2017; Vodopivec-Jamsek et al., 2012). Frequent health education tabling events may combine the cost-effectiveness of leaflets and technology-based dissemination with the benefits associated with intensity (e.g., in-person interaction) and follow-up. Although there has been significant scholarly work regarding the intersection between health and education, there remains a gap in evaluating the effectiveness of educational tabling events at schools. Saksvig et al. (2005) studied a school-based intervention that improved food knowledge and self-efficacy among Native Canadian children. One of the components of the intervention in this study involved information tabling events/booths. However, tabling event/information booths were not a major part of the study’s intervention.

Health education at schools is often thought of as a mandatory presentation that students attend to receive information from the presenters, in contrast to the student-led non-mandatory tabling events that are the focus of this program evaluation. Thus, another area of investigation of this study involved comparing the effect of mandatory versus voluntarily attended educational events. There have been varying findings regarding which method is more effective. One 2004 study found that students who had a mandatory computer learning module viewed it as more useful than students whose module was optional (Garland & Noyes, 2004). On the contrary, a 2015 article concluded that optional learning activities could be beneficial for students (Verkade & Lim, 2015). In contrast, a survey of engineering students found no statistical difference between students taking optional or mandatory courses in leadership in terms of their attitudes toward the courses (Parkinson et al., 2017). Our evaluation examines the survey results from a mandatory presentation on mental health and well-being and from multiple student-led tabling events that had optional attendance by the students.

### Program Description

The mission of the Los Angeles Trust for Children’s Health is “bridging health and education to achieve student wellness” intending to make “a world where every student is healthy and successful” (The LA Trust, 2023). These goals are accomplished by facilitating screenings, health education, and referrals for public school students through wellness centers at schools as well as other programs. Research, advocacy, and policy are also central to its work. Since 1991, The Los Angeles Trust for Children’s Health has supported health centers at Los Angeles (LA) public schools, tackling important issues that affect youth, including substance use prevention, oral health, nutrition, mental health, and Human Papillomavirus infection prevention (The LA Trust, 2023).

One of the initiatives is a student-led program in partnership with the school district called Student Advisory Boards (SABs), which are groups of student health advocates at schools with Wellness Centers who work with adult allies to learn about health topics and educate their peers on important health issues. Adult allies work for The LA Trust for Children’s Health to train and oversee the SABs. This project evaluated the effectiveness of two educational outreach programs: a mandatory health education presentation and educational booths hosted by SAB adult allies working with student advocates across LA public schools. The topics of the health education presentations and educational booths included “Mental Health/Wellness,” “Birth Control/Safe Sex,” “Sexually Transmitted Infections (STIs),” and “Screening, Brief Intervention, and Referral to Treatment (SBIRT)” for substance use. The categories “STIs” and “Birth Control/Safe Sex” were later combined during statistical analysis due to similarity in content. The inclusion of survey data from the mandatory health education presentation served as a baseline that would allow us to compare the impact of the student-led tabling events to a more traditional health education approach.

Even when using well-studied techniques such as peer education, program evaluation is an important way to assess the impact of health education programs and to identify areas for improvement. Moreover, evaluation is necessary when utilizing less-studied health education methods such as optional student-led tabling events. This evaluation addresses one central research question and two sub-questions:

**Research Question 1 (RQ1):** What is the impact of peer-led short-term health education tabling events at schools?
**Research Question 1A (RQ1A):** How does this impact vary based on health topics and grade level?
**Research Question 1B (RQ1B):** Do students view the results of non-mandatory peer-to-peer tabling events and traditional mandatory health presentations differently?

## Method

We analyzed quantitative data from surveys administered at nineteen health education events that The LA Trust for Children’s Health hosted at eight public high schools in Los Angeles County. This included 343 survey responses across these events and schools, although not all students who attended the events completed the survey. These tabling events were hosted by SABs overseen by the adult allies, which are clubs at schools where students work with adult allies to learn about health topics and share health education with their peers. One larger health education presentation event was also evaluated using the same survey. Attendance by the students at this presentation was mandatory whereas the tabling events were completely voluntary and took place during the school day, usually during the students’ lunch break. The design of this study was based in part on Maxwell’s Handbook of Applied Social Research Methods to have an effective program evaluation (Maxwell et al., 2009). The surveys were created using Microsoft Forms, and attentiveness to the protection of private information was enacted in accordance with the ethical standards of social research. All procedures were processed and determined to be exempt by The University of California, Los Angeles (UCLA) Institutional Review Board (IRB).

### Data Collection

Data was collected using surveys previously created by The LA Trust for Children’s Health with questions about the impact of the health education events. The questions can be found in Table 1. Examples of questions included “Did you learn something useful?” or “How likely are you to attend similar events in the future?” Survey questions had five answer options: “Strongly Agree,” “Agree,” “Neither Agree nor Disagree,” “Disagree,” and “Strongly Disagree.” Values were assigned from 5 (*strongly agree*) to 1 (*strongly disagree*). For the last two questions, the four answer options were “Very likely,” “Likely,” “Not Likely,” and “Very Unlikely.” Values were assigned from 4 (*very likely*) to 1 (*very unlikely*).

**Table 1:** Survey Questions

Survey Question	Answer Option (Corresponding Numerical Value)
What grade are you in?	9th 10th 11th 12th
Was this the first time you attended one of our activities/events?	Yes No
I was able to connect with others.	Strongly agree (5) Agree (4) Neither Disagree nor Agree (3) Disagree (2) Strongly disagree (1)
I learned something new/useful to me.	Strongly agree (5) Agree (4) Neither Disagree nor Agree (3) Disagree (2) Strongly disagree (1)
I learned some tips/tools/resources that can strengthen my well-being.	Strongly agree (5) Agree (4) Neither Disagree nor Agree (3) Disagree (2) Strongly disagree (1)
Based on your experience today, how likely are you to attend future activities/events?	Very likely (4) Somewhat likely (3) Not unlikely (2) Very unlikely (1)
Based on your experience today, how likely are you to recommend our activities/events to your friends?	Very likely (4) Somewhat likely (3) Not unlikely (2) Very unlikely (1)

*Note.* Questions were asked to event attendees via Microsoft forms. QR codes to the forms were posted at each event.

The surveys were available via QR codes posted on papers at each event. Students were able to scan the QR code after visiting the tabling event and completed the survey on their phones. To ensure equity, students without a phone were allowed to utilize one of their peer educators’ phones to complete the survey. Students were told to fill out the survey only once. Online surveys accessible via the QR codes were used because phones are widely used by high school students, and students are very comfortable using them in a school setting (Thomas & Muñoz, 2016). In addition, some events distributed stickers or flyers with the QR code for students to complete at a later time. Some schools gave small prizes such as candy to students who completed the survey. The inclusion of the prizes was to increase response rates (Taylor-Powell & Hermann, 2000).

### Data Analysis

Statistical analyses were performed using *R* (version 4.3.0). Chi-square tests of independence, Kruskal–Wallis tests, and Wilcoxon rank sum tests with continuity correction were performed to determine if there were significant differences based on grade level, health topic, whether this was the student’s first attendance at an event, and mandatory versus voluntary tabling events. Specifically, we aimed to further study the research gaps identified by Mellanby et al. regarding the effectiveness of peer-led versus adult-led school health education (Mellanby et al., 2000) by evaluating the impact of student-led health education events. We also aimed to understand which health topics were more popular. All data was kept private and confidential, with password protection.

## Results

In total, there were 343 survey responses from students, with 145 of the responses from the non-voluntary “Mental Health/Wellness” event and 198 responses from voluntary tabling events. Of the surveys, 27% were completed by 9th graders (*N*=93), 19% by 10th graders (*N*=64), 20% by 11th graders (*N*=67), and 35% by 12th graders (*N*=119). Across all *N*=343 survey responses, 26% (*N*=88) of them fell under the category of “Mental Health/Wellness,” 59% (*N*=203) were related to “Safe Sex and STI” events, and 15% *(N*=52) were from the “SBIRT” event type (although the original events and surveys included separate categories for Birth Control/Safe sex and STIs, the categories were combined for statistical analysis). Voluntary and non-voluntary events were analyzed separately, as the methodologies for data collection and recruitment between these types of events were different. The proportion of students in each grade was somewhat evenly distributed, although there was a higher proportion of 12th graders.

Overall, the students had a very positive view of the optional tabling events, similar to the more traditional mandatory health education events. The vast majority stated that they learned something of value and connected with their peers while also saying that they would like to attend similar future events and recommend them to peers. Detailed results for each question and event type are outlined in Tables 1 and 2.

**Table 2:** Survey Responses for the Non-Voluntary Event

Question	Mean response	Standard Deviation
I was able to connect with others.	4.26	0.707
I learned something new/useful to me.	4.24	0.690
I learned some tips/tools/resources that can strengthen my well-being.	4.28	0.618
Based on your experience today, how likely are you to attend future activities/events?	3.72	0.478
Based on your experience today, how likely are you to recommend our activities/events to your friends?	3.69	0.559

*Note.* Mean survey responses and standard deviations were calculated for the non-voluntary health education event and were used to generate the table above. The scale for the first three questions was 1 to 5 while the scale for the latter two questions was 1 to 4.

### Impact on Students

**Figure 1:**
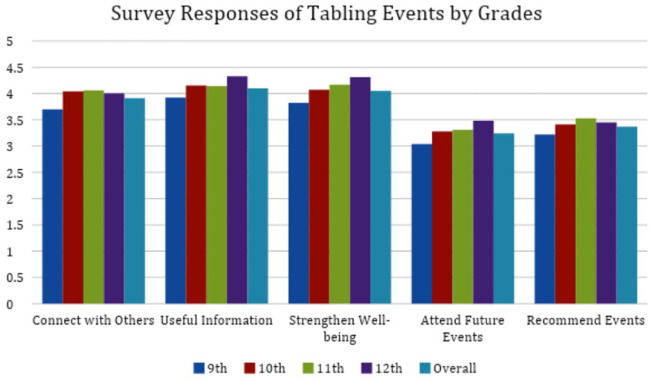
Mean Survey Responses by Grade Level for Voluntary Tabling Events* *Note*. Mean survey responses to each question were calculated for each grade and were used to generate the above graph. *For “Attend Future Events” and “Recommend Events” the scale was 1-4 while the other questions had a scale of 1-5.

The survey responses showed that for all topics, respondents tended to agree that they were able to connect with others, learned something useful, and learned some tips/tools/resources that can strengthen their well-being (see Figure 1 and Table 2). They also were likely to attend future events or recommend similar events to friends. Although still receiving relatively positive feedback, the mental health topic had the lowest average scores for most questions. In addition, “STIs” had the highest average grade level of respondents (11.2), followed by “Birth Control/Safe Sex” (10.7), with “Mental Health/Wellness” (9.9) and “Screening, Brief Intervention, and Referral to Treatment (SBIRT)” (9.9) having the youngest average grade level respondents.

### Likeliness to Attend Again or Recommend to Friends

The students responded that they were likely to attend future events and to recommend the activities to friends, with the vast majority saying that they were likely or very likely. Furthermore, the majority of students at each event were attending for the first time, ranging from 69% for birth control/safe sex to 90% for STIs.

### Voluntary Health Education Tabling Events

Kruskal Wallis tests of survey responses by grade level showed that at least one average rank was different for the Likert-type-scale questions “I learned something new/useful to me” and “I learned some tips/tools/resources that can strengthen my well-being” (respective *p* values = .02 and .02), with “I learned something new/useful to me” having a slightly higher average. Ninth graders also had the lowest mean response scores for all five survey responses, despite having the largest sample size. Among the different health categories, “Mental Health/Wellness” had the lowest mean scores for all five survey questions. In terms of attendance, 141 (71.2%) of the 198 respondents said they were first-time attendees to the Tabling Event. Kruskal Wallis tests of survey response questions by mental health/wellness topic showed that at least one average rank was different for the Likert-type-scale questions “I learned something new/useful to me,” “I learned some tips/tools/resources that can strengthen my well-being,” and “Based on your experience today, how likely are you to attend future activities/events?” (*p* values = .01, .0003, and .001, respectively).

### Mandatory Health Education Presentation Event

A summary of the responses to the non-voluntary health education events can be found in Table 2. For the mandatory mental health education event, Kruskal Wallis tests of survey response questions by grade level showed that at least one average rank was different for the Likert-type-scale questions “I was able to connect with others,” “I learned something new/useful to me,” “I learned some tips/tools/resources that can strengthen my well-being,” and “Based on your experience today, how likely are you to attend future activities/events?” (*p* values = .02, .03, .045, and .01, respectively). The mandatory health education event had significantly higher average responses than did the optional tabling events, to the questions “I was able to connect with others,” “I learned some tips/tools/resources that can strengthen my well-being,” “Based on your experience today, how likely are you to attend future activities/events?,” and “Based on your experience today, how likely are you to recommend our activities/events to your friends?” (*p* values = <.001, .005, <.001, and <.001, respectively; see Table 3).

**Table 3:** Mandatory Versus Voluntary Educational Event Responses

Survey Question	Mandatory Event (*N*=145)	Voluntary Events (*N*=198)	*p* value
First Attendance			
No	10 (6.9%)	57 (28.8%)	<.001
Yes	135 (93.1%)	141 (71.2%)	
Connect With Others			
Mean (*SD*)	4.26 (0.707)	3.91 (0.826)	<.001
Learned Useful Information			
Mean (*SD*)	4.24 (0.690)	4.10 (0.754)	.083
Strengthened Well-being			
Mean (*SD*)	4.28 (0.618)	4.05 (0.763)	.005
Will Attend Future Events			
Mean (*SD*)	3.72 (0.478)	3.24 (0.793)	<.001
Recommend Events to peers			
Mean (*SD*)	3.69 (0.559)	3.37 (0.713)	<.001

*Note.* Mean survey responses were calculated for the voluntary and non-voluntary health education events and compared in the graph above. P-values were calculated to determine the statistical significance of the differences. The scale for the first three questions was 1 to 5 while the scale for the latter two questions was 1 to 4.

## Discussion

Overall, the students had very positive responses toward both the voluntary tabling events and the mandatory presentation, with the vast majority learning something they considered new or useful. The student-led tabling events had similar effectiveness to the traditional mandatory health education presentation, showing the potential for this method of peer-to-peer health education. The students were very likely to attend similar events in the future and recommend the events to others. Notably, “Mental Health/Wellness” had the highest rate of first-time visitors, but tended to have lower scores, although still overall positive results. “STIs” had the highest average age of the attendants followed by “Birth control/safe sex.” Ninth-grade students generally gave lower scores on most topics compared with students in older grades.

These results show that optional health education tabling events, particularly with a peer-to-peer education aspect, at schools can be an effective way of disseminating health information to youth populations, supporting previous literature (Geetha Priya et al., 2019; Saksvig et al., 2005). Thus, booths or tabling events at schools on specific health topics can be used more frequently to educate students about certain health topics. To our knowledge, this is the first evaluation specifically studying school-based health education tabling events. This evaluation can inform future efforts starting student-led health education tabling programs as well as expanding on existing programs.

STIs and safe sex/birth control had the highest average age of attendees, suggesting that students of younger ages may not be the best target of events about these topics. Thus, health educators may consider surveying younger students to see what issues resonate most with them. Other topics such as mental health and substance abuse methods may be more impactful than topics related to sex for 9th and 10th graders. Alternatively, 9th- and 10th-grade students may also be more embarrassed about attending STI or Safe Sex events. If this is the case, more private or anonymous education programs may be more effective for sex-related topics. STIs also tended to have the most positive average responses, suggesting that there is a need among high school students to better understand the risks of having sex.

As mental health is an important issue (Oliveira et al., 2022), it was surprising that it received lower ratings than other topics. This may suggest that education and awareness may not be as effective as more concrete methods that directly provide adolescents with outlets to improve their mental wellness, such as more physical activity opportunities, decreased academic pressure, and later school start times to promote healthier sleeping habits. Students may also be fatigued from hearing so much about mental health without feeling substantial improvements in their own lives or sense of mental well-being. More research should be conducted.

This evaluation was unique from other studies (Coles et al., 2016; Layzer et al., 2017; Saksvig et al., 2005; Salam et al., 2016) in several ways. First, it focused on school-based tabling events rather than a variety of intervention methods, such as classes, social media posts, or workshops. Thus, this evaluation provides support that school-based tabling events are an effective way of providing health education to high school students. Consequently, this type of intervention can be used as an additional tool to address the health knowledge gaps of adolescents.

While adult allies from the LA Trust for Children’s Health were present at the tabling events, much of the education was conducted by high school students themselves. Our evaluation suggests that peers are a good disseminator of health education, supporting Layzer et al., 2017 previous findings on the topic. This is somewhat in contrast to a 2010 study by Rakoczy et al. (2010) that found that children learn better from adults. Most likely, students are able to learn from both peers and adults, but the relative efficacy may depend on the setting and topic. Further research should be done to determine if the subject matter being taught or the location affects the effectiveness of peer-mediated versus adult-mediated health education. Overall, the findings suggest that student-led initiatives have positive results while also giving the students involved an opportunity to improve their leadership and peer-to-peer education skills. Thus, peer education can serve as a model for health promotion in schools.

This evaluation had several limitations. First, the sample size was limited to 343, and all participants were from Los Angeles, mostly from underserved communities, possibly limiting generalizability to other regions. In addition, only five questions were used which were previously developed by The LA Trust for Children’s Health for program evaluation. This limited the information that could be gathered. Future evaluations should utilize follow-up surveys, mixed methods, and/or more extensive survey questions to build on the findings of this paper. Moreover, only four very general health topics (although they were diverse) were investigated despite there being many more potential topics that could have different results. As no other age groups were examined in this study, these results may only apply to high school students. Confounding variables, such as the quality of the educational materials and the effort of the educators could also affect the responses. Finally, while the surveys clearly show that knowledge has been transmitted, it is less certain what the impact of the knowledge will be on health behavior and how long this information will be retained. Consequently, while students may prefer the non-mandatory events, it is possible that the higher-effort presentation event could actually lead to better retention and outcomes. These limitations should be addressed in future studies.

Despite these limitations, this evaluation has several important conclusions that add to the existing health education program evaluation literature and from which recommendations for health organizations and schools can be developed. “Mental Health/Wellness” had the highest percentage of first-time attendees, suggesting that there is high interest in this topic among high school students. However, this topic also had the lowest scores, so current mental health education techniques used at tabling events could be improved. “Birth Control/Safe Sex” had the highest average age of attendees, suggesting that younger students may not be as interested in subjects related to sexuality presented in this manner or may not feel as comfortable accessing this information. The mandatory event at one school and the optional events at other schools had similar average survey responses, although the mandatory event had slightly higher scores. This suggests that there is not a large difference in impact between mandatory and optional health education events. As both had positive survey responses, mandatory and optional education events seem to be viable methods for delivering health information to high school students. In regard to age, this evaluation suggests that younger students, specifically 9th graders, do not find the health education events as impactful as older students as they tend to give lower scores. The most important and most clearly supported conclusion from these surveys is that students who visit health education events at schools are positively impacted by the events and generally view them as helpful and informative.

### Implications for Practice

This program evaluation provides valuable information regarding evidence-based practice, particularly in regard to tabling which is a low-cost way to use peer-to-peer education for health promotion while allowing for more youth involvement in health education. From these conclusions, several recommendations for health organizations and schools were developed. First, the implementation of health education tabling events and presentations at schools, including those with a peer-to-peer education component, should be continued and expanded on due to the positive survey results they received. Involving students in the health education process is an effective and cost-efficient option for increasing youth engagement. Both mandatory and optional health education events can be considered as both had positive perceptions by students. In addition, these organizations and schools should explore possible reasons for the lower impact of mental health events. They should also survey students to discover other topics of interest to high school students while recognizing the importance of mental and sexual health education. This will allow schools and health organizations to identify more health topics of greater interest to their students and to tailor their health education programs to these interests.

### Implications for Research

In addition to practice recommendations, this program evaluation suggests several avenues for future research. More studies should be conducted to further investigate school-based health education events. Specifically, future research should be done into the effectiveness of health education events focused on other health topics, different age groups, and varying regions of the country. In addition, as mental health/wellness received less positive results than other topics, more studies should be conducted to find more effective ways to improve mental health and wellness education, especially given the crisis of mental health issues among adolescents.

Despite the previously mentioned limitations, these findings can have wide-ranging implications. As chronic health problems plague children across the country, health education, particularly through schools, should be a central part of policies aimed at combating these poor health outcomes. This evaluation reinforces the value of hosting health education events to encourage student wellness.

## Supplemental Material

sj-docx-1-hpp-10.1177_15248399241258462 – Supplemental material for Evaluation of Health Education Events With a Peer-to-Peer Component in Public High Schools Supplemental material, sj-docx-1-hpp-10.1177_15248399241258462 for Evaluation of Health Education Events With a Peer-to-Peer Component in Public High Schools by Tai Metzger, Alex Zepeda and Naomi Wilcox in Health Promotion Practice
