# Supplementary material for: Evaluation of Health Education Events With a Peer-to-Peer Component in Public High Schools
Source: Health Promot Pract. 2024 Jun 19;26(4):647–56. doi: 10.1177/15248399241258462 (PMC12149449; doi:10.1177/15248399241258462)
Supplement: sj-docx-1-hpp-10.1177_15248399241258462 – Supplemental material for Evaluation of Health Education Events With a Peer-to-Peer Component in Public High Schools [file sj-docx-1-hpp-10.1177_15248399241258462.docx]

**Supplementary Materials**

**Supplementary A.** Survey responses at voluntary tabling events by grade.

| **Question** | **9**  **(N=74)** | **10**  **(N=46)** | **11**  **(N=36)** | **12**  **(N=42)** | **Overall**  **(N=198)** |
| --- | --- | --- | --- | --- | --- |
| *Was this the first time you attended one of our activities/events?* |  |  |  |  |  |
| No | 13 (17.6%) | 14 (30.4%) | 11 (30.6%) | 19 (45.2%) | 57 (28.8%) |
| Yes | 61 (82.4%) | 32 (69.6%) | 25 (69.4%) | 23 (54.8%) | 141 (71.2%) |
| *I was able to connect with others.* Mean (SD) | 3.70 (0.887) | 4.04 (0.729) | 4.06 (0.791) | 4.00 (0.796) | 3.91 (0.826) |
| Median [Min, Max] | 4.00 [1.00, 5.00] | 4.00 [2.00, 5.00] | 4.00 [2.00, 5.00] | 4.00 [2.00, 5.00] | 4.00 [1.00, 5.00] |
| *I learned something new/useful to me.* |  |  |  |  |  |
| Mean (SD) | 3.92 (0.754) | 4.15 (0.759) | 4.14 (0.833) | 4.33 (0.612) | 4.10 (0.754) |
| Median [Min, Max] | 4.00 [2.00, 5.00] | 4.00 [1.00, 5.00] | 4.00 [1.00, 5.00] | 4.00 [2.00, 5.00] | 4.00 [1.00, 5.00] |
| *I learned some tips/tools/resources that can strengthen my well-being.* |  |  |  |  |  |
| Mean (SD) | 3.82 (0.897) | 4.07 (0.712) | 4.17 (0.609) | 4.31 (0.563) | 4.05 (0.763) |
| Median [Min, Max] | 4.00 [1.00, 5.00] | 4.00 [2.00, 5.00] | 4.00 [3.00, 5.00] | 4.00 [3.00, 5.00] | 4.00 [1.00, 5.00] |
| *Based on your experience today, how likely are you to attend future activities/events?* |  |  |  |  |  |
| Mean (SD) | 3.04 (0.898) | 3.28 (0.720) | 3.31 (0.786) | 3.48 (0.594) | 3.24 (0.793) |
| Median [Min, Max] | 3.00 [1.00, 4.00] | 3.00 [1.00, 4.00] | 3.00 [1.00, 4.00] | 4.00 [2.00, 4.00] | 3.00 [1.00, 4.00] |
| *Based on your experience today, how likely are you to recommend our activities/events to your friends?* |  |  |  |  |  |
| Mean (SD) | 3.22 (0.832) | 3.41 (0.652) | 3.53 (0.506) | 3.45 (0.670) | 3.37 (0.713) |
| Median [Min, Max] | 3.00 [1.00, 4.00] | 3.00 [1.00, 4.00] | 4.00 [3.00, 4.00] | 4.00 [2.00, 4.00] | 3.00 [1.00, 4.00] |

**Supplementary B.** Survey responses for voluntary events

| **Question** | **Mean response** | **Standard Deviation** |
| --- | --- | --- |
| I was able to connect with others. | 3.91 | 0.826 |
| I learned something new/useful to me. | 4.10 | 0.754 |
| I learned some tips/tools/resources that can strengthen my well-being. | 4.05 | 0.763 |
| Based on your experience today, how likely are you to attend future activities/events? | 3.24 | 0.793 |
| Based on your experience today, how likely are you to recommend our activities/events to your friends? | 3.37 | 0.713 |

**Supplementary C**. Mandatory versus optional educational event responses.

|  | **Mandatory**  **(N=145)** | **Optional**  **(N=198)** | **P-value** |
| --- | --- | --- | --- |
| first_attendance |  |  |  |
| No | 10 (6.9%) | 57 (28.8%) | <0.001 |
| Yes | 135 (93.1%) | 141 (71.2%) |  |
| connect_others |  |  |  |
| Mean (SD) | 4.26 (0.707) | 3.91 (0.826) | <0.001 |
| Median [Min, Max] | 4.00 [2.00, 5.00] | 4.00 [1.00, 5.00] |  |
| learn_new |  |  |  |
| Mean (SD) | 4.24 (0.690) | 4.10 (0.754) | 0.083 |
| Median [Min, Max] | 4.00 [2.00, 5.00] | 4.00 [1.00, 5.00] |  |
| learn_wellbeing |  |  |  |
| Mean (SD) | 4.28 (0.618) | 4.05 (0.763) | 0.005 |
| Median [Min, Max] | 4.00 [2.00, 5.00] | 4.00 [1.00, 5.00] |  |
| attend_future |  |  |  |
| Mean (SD) | 3.72 (0.478) | 3.24 (0.793) | <0.001 |
| Median [Min, Max] | 4.00 [2.00, 4.00] | 3.00 [1.00, 4.00] |  |
| recommend_friend |  |  |  |
| Mean (SD) | 3.69 (0.559) | 3.37 (0.713) | <0.001 |
| Median [Min, Max] | 4.00 [1.00, 4.00] | 3.00 [1.00, 4.00] |  |
